# Advancing Cardiovascular Diagnostics: The Expanding Role of CMR in Heart Failure and Cardiomyopathies

**DOI:** 10.3390/jcm14030865

**Published:** 2025-01-28

**Authors:** Antonio Luca Maria Parlati, Ermanno Nardi, Federica Marzano, Cristina Madaudo, Mariafrancesca Di Santo, Ciro Cotticelli, Simone Agizza, Giuseppe Maria Abbellito, Fabrizio Perrone Filardi, Mario Del Giudice, Francesco Ruggiero Annunziata, Isabel Martone, Maria Prastaro, Stefania Paolillo, Pasquale Perrone Filardi, Paola Gargiulo

**Affiliations:** 1 Department of Advanced Biomedical Sciences, University of Naples Federico II, 80131 Naples, Italy; 2 Cardiology Unit, Department of Health Promotion, Mother and Child Care, Internal Medicine and Medical Specialties, University Hospital P. Giaccone, University of Palermo, 90127 Palermo, Italy

**Keywords:** cardiac magnetic resonance, heart failure, cardiomyopathies

## Abstract

Cardiovascular magnetic resonance (CMR) imaging has become a cornerstone in the diagnosis, risk stratification, and management of cardiovascular disease (CVD), particularly heart failure (HF) and cardiomyopathies. Renowned as the gold standard for non-invasive quantification of ventricular volumes and ejection fraction, CMR delivers superior spatial and temporal resolution with excellent tissue–blood contrast. Recent advancements, including T1, T2, and T2* mapping, extracellular volume quantification, and late gadolinium enhancement, enable precise tissue characterization, allowing early detection of myocardial changes such as fibrosis, edema, and infiltration. These features provide critical insights into the pathophysiological mechanisms underlying HF phenotypes and diverse cardiomyopathies, enhancing diagnostic accuracy and guiding therapeutic decisions. This review explores the expanding role of CMR in CV disease, highlighting its diagnostic value in HF and in several cardiomyopathies, as well as its contribution to improving patient outcomes through detailed tissue characterization and prognosis.

## 1. Introduction

Cardiovascular magnetic resonance (CMR) imaging has become an essential tool throughout various stages of diagnosing and treating different cardiovascular (CV) diseases, as highlighted in Table 1. Although echocardiography remains the cornerstone for the diagnosis of CV diseases, due to its accessibility, safety, and cost-effectiveness, CMR is widely regarded as the gold standard for non-invasive quantification of ventricular volumes and ejection fraction (EF), offering unmatched temporal and spatial resolution, along with clear tissue–blood differentiation [1]. Over the years, CMR has significantly increased our understanding of CV diseases’ pathogenesis. Technological and software innovations have led to the development of sophisticated techniques that significantly enhance risk stratification, diagnostic accuracy, and prognostic evaluation. Techniques such as extracellular volume (ECV) mapping and T1, T2, and T2* imaging provide detailed tissue characterization, enabling the detection of myocardial changes associated with heart failure (HF) and various cardiomyopathies. Furthermore, late gadolinium enhancement (LGE) offers invaluable structural, functional, and prognostic insights across a spectrum of cardiovascular conditions. These findings often reflect alterations in the myocardial interstitium, such as fibrosis, amyloid accumulation, or edema, as well as intracellular changes within cardiomyocytes, including fat deposition, iron overload, or myocardial infarction [2,3].

**Figure 1:**
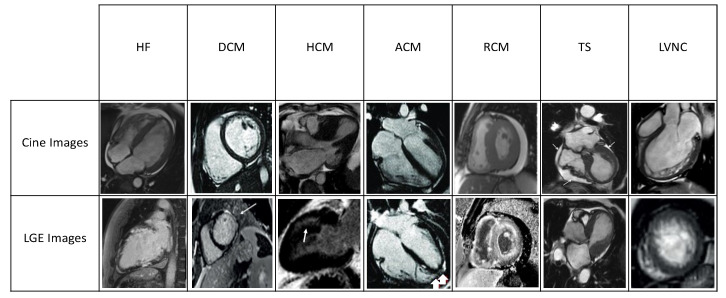
Cardiomyopathies seen through cardiac MRI. HF: heart failure; DCM: dilated cardiomyopathy; HCM: hypertrophic cardiomyopathy; ACM: arrhythmogenic cardiomyopathy; RCM: restrictive cardiomyopathy; TS: Takotsubo syndrome; LVNC: left ventricular noncompaction.

This review explores the transformative role of CMR in CV diseases, focusing on its diagnostic utility in HF and multiple forms of cardiomyopathy (Figure 1).

## 2. CMR in HF

HF is a condition characterized by signs of inadequate cardiac output and/or volume overload, resulting from the heart’s inability to meet the physiological demands of other organs [4]. As HF often arises from diverse underlying conditions, personalized treatment approaches are increasingly essential. HF is typically categorized into three phenotypes according to left ventricular EF: HF with reduced EF (HFrEF, EF ≤ 40%), HF with mildly reduced EF (HFmrEF, EF 41–49%), and HF with preserved EF (HFpEF, EF ≥ 50%). Although treatment decisions are still predominantly guided by EF, this parameter has notable limitations. Indeed, EF values can fluctuate depending on the imaging modality employed, and, at higher levels, EF may lack the sensitivity to detect early left ventricular systolic dysfunction or account for variations in loading conditions [5,6]. Echocardiography is considered the first line imaging modality for HF because it is a fast, cheap, and widely available imaging technique, useful for estimating volumes and both systolic and diastolic function of the left ventricle. However, the quality of echocardiographic images may be limited by factors such as obesity, lung interposition, or chest conformation. Moreover, the visualization of the right chambers and the myocardial fibrosis is limited. Although more expensive and less accessible, CMR is considered the gold standard, due to its capacity to obtain more accurate measures of the volumes and function of both the right and left ventricles, to identify myocardial fibrosis patterns, and to distinguish ischemic versus non-ischemic causes. A comprehensive comparison between echocardiography and CMR in the assessment of HF is presented in Table 2. The superior spatial and temporal resolution, combined with the absence of ionizing radiation, makes CMR a safe and precise tool for evaluation of cardiac anatomy and physiology. Modern CMR-compatible cardiac devices further expand its applicability, allowing CMR to be used in most patients with appropriate precautions [7]. CMR is highly effective in assessing EF and wall motion abnormalities due to its reproducibility and spatial and temporal resolution, along with its unobstructed visualization of the heart. Modern cine imaging techniques enable comprehensive imaging of the heart in multiple planes (short and long axis) with high contrast between blood and myocardium, spatial resolution ≤1.5 mm^2^, and temporal resolution ≤45 ms [8]. These features allow the assessment of ventricular size, shape, systolic function, cardiac mass, and stroke volume. Furthermore, CMR can capture pericardial images and detect myocardial edema with T2-weighted sequences. Gadolinium diffusion is increased in both acute and chronic cardiac conditions as a consequence of cell injury and extracellular fibrosis [9]. Gadolinium-based contrast agents are unable to penetrate intact cell membranes, while injured cells exhibit slower gadolinium washout, thus enabling differentiation between normal and pathological myocardium. Healthy myocardium appears dark on LGE images, while the injured tissue remains hyper-enhanced and appears white [9]. LGE patterns provide valuable diagnostic and prognostic insights into the ischemic and non-ischemic etiology of HF. For ischemic conditions, damage typically begins in the subendocardium and progresses outward. In contrast, non-ischemic cardiomyopathies display more variable LGE patterns [10,11]. However, in diffuse diseases, LGE may have lower sensitivity due to reduced gadolinium uptake in areas with diminished blood flow. Techniques such as pharmaceutical vasodilation can identify stress-induced perfusion deficiencies by enhancing coronary artery flow [12]. Given EF shortcomings, alternative markers including left ventricular strain are gaining prominence. Strain measures myocardial deformation in specific dimensions (usually radially, longitudinally, and circumferentially) thus enabling the detection of localized myocardial contractile dysfunction with increased sensitivity, including cases where EF is preserved. Reduced global longitudinal strain (GLS) measured by CMR correlates with increased cardiovascular complications, decreased functional capacity, and higher mortality risk [13]. CMR-derived strain measurements can also predict abnormalities in left ventricular relaxation as measured invasively [14]. Assessing right ventricular function, which is often challenging with echocardiography, is more feasible with CRM. Using short-axis imaging, right ventricular volumes and contractility can be accurately measured alongside left ventricular assessments. Impaired right ventricular EF on CMR has been associated with worse outcomes, including higher rates of CV death and HF hospitalization, regardless of left ventricular EF [15]. CMR can also assess right ventricular strain, which is a predictor of poor outcomes in HF and reduced responsiveness to cardiac resynchronization therapy (CRT) [16,17]. Alterations in CRM markers of fibrosis have been identified as potential imaging indicators for future heart failure events [18,19]. In this context, several studies have investigated the relationship between circulating cardiac biomarkers, such as cardiac troponins and natriuretic peptides, and CMR markers. Yang et al. have demonstrated that elevated non-invasive measurements of ECV via CMR imaging are linked to incident HF outcomes in patients with elevated high-sensitivity troponin T levels and increased N-terminal pro B-type natriuretic peptide (NT-proBNP) serum levels [20]. In addition, Iyer et al. showed that GLS measurement through CMR and soluble ST2 plasma levels provides independent and incremental prognostic value in predicting adverse outcomes in HF patients with diabetes, compared to those without [21]. Therefore, integrating imaging markers with circulating biomarkers could play a valuable role in enhancing risk stratification and providing prognostic information in patients with HF. Additionally, growing evidence highlights a critical role of CMR in uncovering the pathophysiologic mechanisms of HFpEF, such as myocyte hypertrophy, interstitial fibrosis, coronary microvascular and macrovascular dysfunction, and metabolic derangements [22]. Thanks to its ability to identify the HFpEF phenotypes, CMR offers a valid instrument to establish the prognosis and guide more targeted therapeutic interventions, improving overall clinical outcomes [23,24]. Kanagala et al. observed that both focal and diffuse fibrosis are more prevalent in HFpEF patients compared to healthy subjects [25]. Greater left ventricular mass and impaired longitudinal function were associated with greater ECV changes [26]. Myocardial fibrosis, detected by LGE and ECV quantification, is associated with a significantly increased risk of hospitalizations and cardiovascular death in patients with HFpEF [27].

Signs of right ventricular dysfunction on CMR in HFpEF patients are associated with increased mortality and pulmonary hypertension [28]. CMR is also well-suited to evaluate primary structural disorders of the right ventricle in HF, such as congenital heart defects and arrhythmogenic right ventricular cardiomyopathy [29,30].

CMR is increasingly applied to evaluate diastolic function. Rathi et al. have shown that CMR phase contrast imaging can measure mitral inflow and pulmonary vein velocities, though values may be underestimated compared to Doppler echocardiography [31]. Furthermore, Su et al. demonstrated that diffuse myocardial fibrosis plays a unique role in the diastolic function, with higher values of ECV correlating significantly with a diastolic dysfunction [32]. Emerging CMR techniques, such as 4D flow imaging, offer promising tools for detecting diastolic dysfunction and pulmonary artery flow [33,34]. These techniques also quantify flow abnormalities, including valve regurgitation severity [35]. A notable application of 4D flow imaging is the non-invasive measurement of intracardiac pressure gradients through vortex pattern analysis and relative pressure estimates. For example, pulmonary artery vortices identified by 4D flow have been strongly correlated with invasive measures of mean pulmonary artery pressure in patients undergoing right heart catheterization for suspected pulmonary hypertension [36]. Furthermore, Kim et al. showed that 4D pulmonary flow analysis is crucial for understanding pulmonary hypertension etiology [37].

Technological advancements, such as real-time free-breathing cine imaging, now enable the acquisition of a full cardiac cycle in 3D in under 24 s with image quality comparable to traditional 2D sequences. These developments continue to enhance the diagnostic and prognostic capabilities of CMR, solidifying its role as a cornerstone in HF management.

## 3. CMR in Dilated Cardiomyopathy

Dilated cardiomyopathy (DCM) is a heart muscle disorder primarily characterized by the enlargement and dysfunction of one or both ventricles, independent of ischemic heart disease. Among diagnostic modalities, CMR has become the gold standard method for evaluating biventricular volumes, mass, and EF, using cine steady-state free-precession (SSFP) sequences. Additionally, CMR plays a critical role in detecting myocardial fibrosis through LGE sequences [38]. Approximately 25–50% of DCM patients exhibit LGE, which typically displays a patchy, midwall, or subepicardial distribution. Since Assomull et al.’s landmark study in 2006, midwall fibrosis detected by LGE has been recognized as a prognostic marker for adverse outcomes, including all-cause mortality, CV mortality, and sudden cardiac death (SCD) [39,40]. A recent study enrolling 399 DCM patients with EF > 40% demonstrated a striking 9-fold increased risk of SCD or aborted SCD in those with midwall LGE compared to those without [41]. Importantly, LGE burden stratifies risk independently of EF or inducible ventricular tachycardia. Myocardial LGE burden >5% is associated with a 20% annual risk of death or major arrhythmia, whereas the absence of LGE correlates with a 3% annual risk [42]. Distinct LGE locations, patterns, or extents impart varying prognostic implications. The absence of LGE also predicts a higher likelihood of reverse remodeling under optimized medical therapy [43], while the presence and extent of transmural LGE in the lateral left ventricular wall indicate a poor response to CRT [44]. Considering recent findings, particularly from the DANISH trial, the assessment of LGE in patients with DCM has become increasingly important. The DANISH trial demonstrated that implantable cardioverter-defibrillator (ICD) therapy in symptomatic patients with a left ventricular ejection fraction (LVEF) ≤ 35% did not improve overall mortality in DCM, primarily because most patients died from HF, not arrhythmic events. ICDs were shown to reduce the risk of SCD but did not affect mortality related to HF [45]. The lack of improvement in overall mortality due to heart failure highlights the need for more precise patient selection when considering ICD implantation. Recent research has proposed a multi-parameter approach to enhance the selection process for ICD therapy, helping to identify patients who would derive the greatest benefit [46]. Among the various invasive and non-invasive markers of arrhythmic risk, LGE-CMR has emerged as one of the most promising [47]. Given this, the latest European guidelines on SCD have incorporated LGE assessment as a potential criterion for ICD implantation in DCM patients [48]. In addition to the standard class IIa indication for ICD implantation in symptomatic patients with NYHA class II–III and LVEF ≤ 35% despite three months of optimal medical therapy, the guidelines also recommend ICD implantation for patients with an LVEF < 50% and at least two risk factors, such as syncope, LGE presence on CMR, inducible sustained monomorphic ventricular tachycardia (SMVT) during programmed electrical stimulation (PES), or pathogenic mutations in genes like LMNA, PLN, FLNC, and RBM20 [48,49]. Furthermore, LGE findings could guide medication titration. Patients with extensive myocardial fibrosis might benefit from more aggressive up-titration of HF medications to reduce ongoing remodeling. This personalized approach can enhance the effects of pharmacological therapy, potentially preventing further myocardial deterioration [43]. In the context of CRT, LGE also plays a key role in predicting the likelihood of response. Leyva et al. showed that optimal placement of the left ventricular lead, away from areas of scarred myocardium, leads to a better response to CRT [44]. In contrast, pacing scarred tissue was linked to the worst outcomes, including higher rates of pump failure and sudden cardiac death [44]. Therefore, assessing LGE is crucial when considering CRT implantation, as it helps determine the optimal lead placement, improving the mechanical benefits of the therapy and ultimately enhancing patient outcomes. Recent findings suggest that GLS more strongly correlates with outcomes such as cardiac death and heart transplantation than left ventricular EF or natriuretic peptides [50]. Furthermore, CMR-derived assessment of right ventricular systolic dysfunction has emerged as a key predictor of all-cause mortality and the need for cardiac transplantation [51].

## 4. CMR in Hypertrophic Cardiomyopathy

Hypertrophic cardiomyopathy (HCM) stands as the prevailing genetic heart disorder, affecting approximately one in 200 to 300 individuals [52]. Although it remains the primary screening tool for HCM, CMR offers superior sensitivity, particularly in cases where echocardiography encounters technical limitations in evaluating hypertrophy. This advantage is especially evident in detecting atypical patterns, myocardial crypts, papillary muscle abnormalities, and apical ventricular aneurysms [53,54]. CMR plays a pivotal role in differentiating myocardial hypertrophy caused by other conditions, including athlete’s heart [55], hypertensive heart disease [56], and infiltrative disorders like amyloidosis and Fabry’s disease [57,58]. The identification of myocardial fibrosis through LGE imaging is a common finding in HCM, affecting up to 80% of patients [59]. In a recent multicenter study, Chan et al. demonstrated that fibrosis extending over 15% of left ventricular mass is associated with a twofold increase in the risk of SCD, even in the absence of other risk factors [60]. The detected myocardial fibrosis often displays a multifocal and irregular distribution, deviating from coronary anatomy [61,62]. Scar heterogeneity [63], impaired GLS [64], myocardial edema [65], native T1 [66], and ECV mapping [67] are emerging as additional prognostic markers in HCM patients. Notably, native T1 mapping has proven effective in distinguishing HCM from hypertensive heart disease [66]. ECV is particularly useful, as it is elevated not only in phenotypically positive cases of HCM but also in individuals who are genotype-positive but phenotype-negative [67]. Myocardial edema, observed in 20–40% of HCM patients using T2-weighted sequences, frequently coincides with areas of LGE [65,68].

## 5. CMR in Arrhythmogenic Cardiomyopathy

Arrhythmogenic cardiomyopathy (ACM) is a genetic heart disorder associated with ventricular arrhythmias and a significant risk of SCD, particularly among young individuals. Initially recognized as a condition involving only the right ventricle, the expanded use of CMR has facilitated the identification of the biventricular and left ventricular-dominant subtypes, affecting the myocardium of both ventricles and solely the left ventricle, respectively [69]. ACM typically progresses through several stages, spanning from the pre-clinical phase to the manifest phase, in which various clinical scenarios may arise, such as dyspnea, chest pain, syncope, sustained ventricular tachycardia, and ventricular fibrillation, potentially leading to cardiac arrest [70]. CMR is highly valuable for studying all phases of ACM natural history, offering high accuracy and reproducibility in evaluating chamber volumes and distinguishing between genuine cases and phenocopies [71]. In patients with ACM, LGE is particularly useful for visualizing the characteristic lesions of the condition, including the fibro-fatty replacement of the ventricular myocardium. Moreover, LGE can help in categorizing the phenotypic variants of ACM and in stratifying arrhythmic risk, especially in left-dominant forms [30,72]. However, LGE sensitivity varies in right ventricular-dominant ACM, with positive findings detectable in only 30–70% of patients [73]. This variability may result from the limited resolution of LGE techniques for the thin right ventricle wall, often obscured by hyperintense signals from blood and adjacent adipose tissue. T2-weighted sequences for detecting myocardial edema are not routinely recommended, except during the acute phases of the disease, such as those observed in children with desmoplakin gene mutations [74,75]. While CMR is integral to ACM diagnosis, it should not be used in isolation. Instead, it should complement family history, genetic testing, arrhythmic evaluations, and electrocardiography (ECG) findings. Genetic testing has emerged as a crucial component in diagnosing ACM, aiding in the identification of pathogenic variants linked to disease progression and arrhythmic risk. Combining genetic data with CMR findings enhances diagnostic accuracy and risk stratification. Additionally, incorporating genetic findings may help differentiate ACM from phenocopies, improving the specificity of diagnosis and guiding family screening initiatives. This multidisciplinary approach not only aids in excluding conditions that mimic ACM but also contributes to effective risk stratification and tailored management strategies. Indeed, a previous study by te Riele et al. highlighted the significance of a strategic combination of CMR with ECG and Holter monitoring results. This approach proved worthy in identifying ACM patients at a heightened risk of arrhythmias [76]. It is essential to note that CMR findings, when isolated from concurrent ECG, arrhythmic, and genetic information, lack conclusiveness in ACM diagnosis and may not provide meaningful insights for arrhythmic risk stratification.

## 6. CMR in Restrictive Cardiomyopathy

Restrictive cardiomyopathy (RCM) is a clinical condition, marked by an impairment of diastolic function, resulting from altered ventricular filling dynamics. This leads to increased ventricular end-diastolic pressures and atrial dilation, while systolic function is typically preserved [77]. RCM encompasses various etiological categories, including idiopathic, familial, and secondary forms related to systemic disorders such as cardiac amyloidosis (CA), which represents the most common cause of RCM. CA occurs when amyloid fibrils accumulate in the extracellular space of the heart muscle, due to the misfolding of specific proteins. Amyloid deposition typically originates in the subendocardium and progressively infiltrates the myocardium, filling the spaces between muscle fibers. In some cases, the primary site of amyloid deposition may be the pericardium, excluding the endomyocardium [78]. The main types of CA are transthyretin amyloidosis (ATTR), which is common in older adults and can be present in both acquired and hereditary forms, and monoclonal immunoglobulin light-chain amyloidosis (AL), which is an acquired form. The typical pattern of CA at CMR reveals LGE throughout both ventricles, with prominent subendocardial enhancement. This presentation is characterized by a distinctive zebra-stripe pattern, affecting the subendocardium of both ventricles while sparing the interventricular septum [79]. Other manifestations include patchy transmural enhancement and atrial wall involvement [80,81]. Given the diverse LGE patterns, integrating LGE with other techniques, such as T1 mapping and ECV quantification, is essential for accurate diagnosis and risk stratification. T1 mapping has been shown to be advantageous for the detection of early cardiac involvement, even before abnormalities become apparent on LGE imaging. This technique is especially helpful in patients with severe kidney impairment, where gadolinium-based contrast agents are contraindicated [82]. Furthermore, recent studies have highlighted the high sensitivity of T1 mapping in diagnosing AL-type cardiac amyloidosis and its ability to distinguish between AL and ATTR subtypes [83]. In a comparative study by Banypersad et al., assessing the ECV fraction in 60 patients with CA and 82 healthy controls, mean ECV was significantly higher in patients compared to healthy controls (0.25 vs. 0.40, *p* < 0.001). ECV also correlated with smaller QRS voltages (R = 0.57, *p* < 0.001) and poorer 6-min walk test performance (R = 0.36, *p* = 0.03) [84]. Both ECV and native T1 mapping have proven to be reliable prognostic indicators in cardiac amyloidosis, aiding in treatment planning and outcome prediction [85].

## 7. CMR in in Left Ventricular Noncompaction

Left ventricular noncompaction (LVNC) is an underdiagnosed cardiomyopathy characterized by abnormal trabeculations in the left ventricle, with an increased risk of HF and thromboembolic events. The overlap of LVNC features with other cardiac conditions, makes its diagnosis challenging. Petersen et al., comparing the CMR of 70 healthy controls and 107 patients with cardiac disease, including seven with a prior diagnosis of LVNC, proposed a CMR diagnostic criterion, which is a noncompacted-to-compacted layer ratio of the left ventricle greater than 2.3, measured in end-diastole [86]. Nevertheless, Kawel et al, applying this criterion in 1000 participants from the Multi-Ethnic Study of Atherosclerosis cohort, showed that up to 43% participants met the imaging criteria for LVNC, highlighting potential overdiagnosis [87]. In another study, Jacquier et al. demonstrated that a trabeculated left ventricular mass exceeding 20% of total left ventricular mass was highly sensitive (93.7%) and specific for LVNC when compared to DCM, HCM, and healthy controls [88]. Nucifora et al., analyzing the prevalence and extension of LGE in 42 LVNC patients, observed LGE in only 23 patients, making it specific but not universally sensitive for LVNC diagnosis [89]. However, Andreini et al. found LGE to be a strong independent predictor of adverse events in 113 LVNC patients over a 4-year follow-up. Multivariate analysis revealed that LGE, alongside left ventricular dilation, significantly correlated with cardiac events [HR (95% CI): 4.015 (1.64–9.828), *p* = 0.002], highlighting its prognostic value [89,90].

## 8. CMR in Takotsubo Syndrome

Takotsubo syndrome (TS), commonly known as stress-induced cardiomyopathy, is an acute HF condition triggered by intense emotional or neurological stress. Echocardiography plays a key role in diagnosing TS, revealing a distinct left ventricular dysfunction pattern known as apical ballooning [91]. This dysfunction typically affects the distal portion of the left ventricle. Unlike ischemic damage, TS encompasses a myocardial region that extends circumferentially, beyond the distribution of a single coronary vessel. In cases of multivessel disease or atypical TS presentations, echocardiography may yield ambiguous findings, necessitating CMR for a definitive diagnosis. CMR excels in TS diagnosis with its superior anatomical and functional imaging capabilities. Cine sequences allow precise evaluation of contractile dysfunction, emphasizing the apical ballooning characteristic of TS [92]. Advanced CMR features, such as tissue tracking, enable objective strain measurements and identification of atypical patterns, including midventricular or basal ballooning and biventricular involvement [93]. Moreover, the analysis of myocardial T2-weighted imaging in TS patients often reveals diffuse transmural edema, which is a distinctive pattern distinguishing TS from other conditions such as myocarditis, which often displays localized subepicardial or mid-myocardial edema. Additionally, in TS, the edema involves multiple regions and is not restricted to a single vascular territory, unlike myocardial infarction, where edema is confined to the area supplied by the affected coronary vessel [94]. These capabilities make CMR indispensable in differentiating TS from other myocardial diseases and in guiding patient management.

## 9. CMR in Rare Cardiomyopathies

In addition to the more well-known cardiomyopathies, there are other less recognized and diagnosed forms that are gaining increasing importance due to CMR. An example is iron overload cardiomyopathy. This cardiomyopathy develops in individuals with genetic hemochromatosis or secondarily to excessive iron accumulation from repeated blood transfusions. Myocardial iron deposition alters T2 relaxation times, enabling the detection of cardiac siderosis. Studies have established a direct correlation between lower T2 values, higher myocardial iron content, and an elevated risk of ventricular arrhythmias. Consequently, specific T2 thresholds are now employed to diagnose iron overload cardiomyopathy, guide the initiation of iron chelation therapy, and assess treatment response [94]. Another example is Anderson–Fabry disease (AFD), a rare X-linked hereditary condition resulting from a partial or complete deficiency of the enzyme α-galactosidase A. The lack of this enzyme results in the accumulation of glycosphingolipids in tissues such as cardiac muscle cells and the coronary vasculature. The hallmark CMR features of AFD include concentric left ventricular hypertrophy and a distinctive non-ischemic pattern of LGE, predominantly affecting the basal inferolateral segment of the LV [95,96]. Additionally, intracellular sphingolipid accumulation results in a characteristic reduction of native T1 relaxation times, allowing early detection even before hypertrophy develops and distinguishing AFD from other hypertrophic cardiomyopathies, which typically show elevated T1 values [58]. T2 mapping has also proven useful in identifying myocardial inflammation, a factor believed to drive disease progression [97].

## 10. CMR Limitations

**Figure 2:**
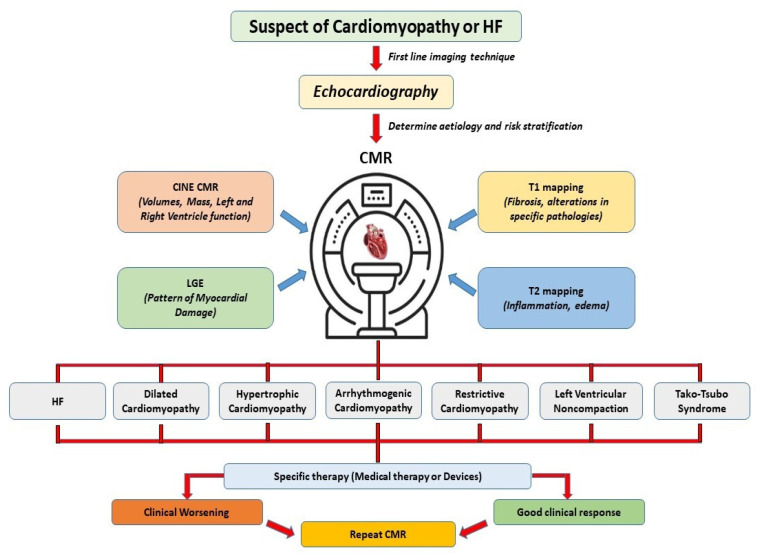
Impact of CMR on clinical decision-making. CMR: cardiac magnetic resonance; HF: heart failure; LGE: late gadolinium enhancement.

Although CMR is a versatile and highly accurate imaging modality, several practical limitations affect its use. Compared to cardiac ultrasound and computed tomography (CT) scans, CMR examinations are time-consuming [98]. While routine studies incorporating perfusion and late enhancement sequences may be completed within 25–30 min, patient preparation, positioning, and entry/exit times extend the total duration. In complex cases or patients with arrhythmias or limited cooperation during breath-holding, examination times may be significantly prolonged. Many standard CMR protocols depend on breath-holding to reduce motion artifacts, and image quality relies heavily on patients’ ability to perform multiple breath-holds of 5–10 s [99]. When patients cannot comply, reduced image quality is expected. Another critical limitation is accessibility. The high cost of CMR equipment, along with maintenance and the requirement for specialized training, limits its availability, especially in resource-limited settings. The expense of CMR can significantly exceed that of echocardiography, posing economic barriers to routine use. Moreover, the need for specialized technicians and cardiologists trained in CMR image acquisition and interpretation restricts broader adoption. Contraindications present additional challenges. Although advances in device technology have enabled scanning of patients with MRI-conditional pacemakers and defibrillators, non-conditional devices, abandoned leads, or epicardial leads still require strict protocols and specialized expertise to minimize risks [100]. Claustrophobia, clinical instability, and pregnancy in the first trimester are relative contraindications, with sedation or alternative imaging required in some cases [101]. Gadolinium-based contrast agents, crucial for LGE and perfusion imaging, pose a small but serious risk of nephrogenic systemic fibrosis in patients with advanced renal dysfunction (GFR < 30 mL/min/1.73 m^2^) [102]. To mitigate these risks, clinicians should consider non-contrast sequences when diagnostic accuracy is preserved or, if contrast is indispensable, use the lowest effective dose of stable agents following shared decision-making with the patient [103]. Given the limitations and contraindications of CMR, the adoption of a multimodal imaging approach has become essential. This strategy enables clinicians to mitigate the individual limitations of each modality while capitalizing on their unique strengths (Table 3). By integrating the complementary information from these techniques, multimodal imaging enhances diagnostic accuracy, guides personalized therapeutic decisions, and optimizes patient management, particularly in complex cases where a single modality may not be sufficient (Figure 2).

## 11. Conclusions

In recent years, despite certain limitations, CMR has emerged as a cornerstone non-invasive tool for diagnosing, assessing risk, and managing HF and cardiomyopathies. Its unparalleled precision in quantifying chamber dimensions, volumes, and cardiac function has significantly enhanced clinical capabilities. Beyond structural evaluation, CMR’s advanced tissue characterization techniques provide critical insights into the underlying pathophysiology of various cardiomyopathies. These features make CMR indispensable in distinguishing between ischemic and non-ischemic etiologies, assessing prognosis, and tailoring individualized treatment strategies. When combined with patient history, clinical observations, ECG, echocardiography, and laboratory findings, CMR serves as a comprehensive and integral component of the diagnostic and therapeutic workflow. This multidimensional approach not only improves diagnostic accuracy but also empowers clinicians to make more informed decisions that positively impact patient outcomes. Looking ahead, the future of CMR holds exciting potential. Emerging technologies, such as real-time imaging and 4D flow techniques, promise to further enhance diagnostic precision.

Additionally, the integration of novel biomarkers and advanced artificial intelligence-driven analysis may unlock new opportunities for early disease detection, personalized treatment, and improved prognosis. Continued research and innovation in these areas will ensure that CMR remains at the forefront of cardiovascular imaging, transforming care for patients with HF and cardiomyopathies.

## Figures and Tables

**Table 1:** Added value of cardiac MRI.

	Role of Cardiac MRI on Clinical Decision-Making
**Gold standard for HF** **imaging**	Cardiac chambers volume, EF, LV and RV mass HF etiology Intraventricular thrombus research
**Diagnostic classification of cardiomyopathies**	Cardiac chambers volume, EF, LV and RV mass Ischemic vs. non-ischemic cardiomyopathy LVH differential diagnosis Right heart disease (ARCV/congenital heart disease) Abnormal ECG findings in athletes Intraventricular thrombus research
**Prognostic stratification of cardiomyopathies**	Cardiac chambers volume, EF of LV and RV LGE quantification ECV quantification
**Therapeutic choices in cardiomyopathies**	Revascularization vs. medical therapy in IHD (ischemia and viability with pharmacological stress, LGE extension) ICD implant (cardiac chambers volume, EF of LV and RV, LGE extension) CRT implant (LGE localization and extension)

HF: heart failure; ARVC: arrhythmogenic right ventricular cardiomyopathy; CRT: cardiac resynchronization therapy; EF: ejection fraction; ICD: implantable cardioverter defibrillator; IHD: ischemic heart disease; LGE: late gadolinium enhancement; LV: left ventricular; LVH: left ventricular hypertrophy; MRI: magnetic resonance imaging; RV: right ventricular.

**Table 2:** Comparison between echocardiography and CMR in the assessment of HF.

Characteristic	Echocardiography	Cardiac Magnetic Resonance
**Ventricular function**	Good for assessing ejection fraction and global function	Excellent visualization of ventricular function
**EF measurement**	Accurate, but image quality can be compromised by poor acoustic window	Precise and reliable EF measurement using ventricular volumes (volumetric segmentation) without acoustic window dependency
**Ventricular volume assessment**	Good, but may be less accurate in patients with obesity or suboptimal acoustic windows	Highly accurate in measuring ventricular volumes, especially when echocardiography is unclear
**Myocardial fibrosis visualization**	Limited	Detailed assessment of myocardial fibrosis using LGE, essential in advanced HF or ischemic/non-ischemic forms
**Identification of HF cause**	Useful for identifying ischemic and valvular causes	Detailed analysis of myocardial structure, useful for distinguishing ischemic vs. non-ischemic causes
**Diastolic dysfunction assessment**	Indirect evaluation using Doppler velocities but limited in advanced staging	Detailed analysis of diastolic function through advanced sequences measuring myocardial stiffness and distensibility
**Identification of ischemic or infarcted areas**	Useful with stress echocardiography, but less sensitive compared to CMR	Excellent for detecting ischemic and infarcted areas, even in the absence of symptoms or positive stress tests
**Right heart imaging**	Difficult to obtain clear right heart images with technical challenges or abnormal morphology	Excellent for right heart evaluation, essential for assessing right heart function and size in right-sided heart failure or pulmonary-related conditions
**Acoustic window**	Effectiveness reduced in obese patients, elderly patients, or those with poor acoustic windows	Not affected by acoustic window quality, making it particularly useful in obese patients or those with other technical difficulties
**Examination time**	Fast (15–30 min)	Long (30–60 min) for full assessment and high-quality imaging
**Accessibility and cost**	Widely available, cheap, and accessible	Expensive, less accessible, specialized equipment required

CMR: cardiac magnetic resonance; EF: ejection fraction; HF: heart failure; LGE: late gadolinium enhancement.

**Table 3:** Strengths and weaknesses of CMR compared to other imaging techniques in heart failure and different cardiomyopathies.

Cardiomyopathies	Strengths of CMR	Weaknesses of CMR	Strengths of Other Techniques	Weaknesses of Other Techniques
**Heart Failure**	- Precise measures of volumes and left and right ventricular function - LGE for fibrosis - Edema evaluation with T2 mapping - Differential diagnosis	- High cost and duration of the exam - Availability - Contraindications with metal devices - Use of contrast - Low quality in arrhythmias of poor breath-holding ability	**Echocardiography:** - Accessibility - Low cost - Evaluation of diastolic function - Main imaging technique during follow-up	**Echocardiography:** - Less useful in patients with a poor acoustic window - Operator dependency
			**CT:** - Exclusion of coronary artery disease	**CT:** - Radiation exposure - Does not evaluate edema or fibrosis - Low quality in arrhythmias
			**Nuclear imaging:** - Evaluation of myocardial perfusion and metabolism	**Nuclear imaging:** - Radiation exposure - Limited in tissue characterization
**Dilated Cardiomyopathy**	- Precise measures of volumes and left and right ventricular function - LGE for necrosis and fibrosis - Differential diagnosis	- High cost and duration of the exam - Availability - Contraindications with metal devices - Use of contrast - Low quality in arrhythmias of poor breath-holding ability	**Echocardiography:** - Accessibility - Low cost - Main imaging technique during follow-up	**Echocardiography:** - Lower volume accuracy - No tissue characterization - Operator dependency
**Hypertrophic Cardiomyopathy**	- Accurate wall thickness and volumes measures - LGE for fibrosis - Risk stratification - Accurate measurement of the LVOT geometry - Differential diagnosis (in complex anatomy cases, MRI offers greater diagnostic clarity)	- High cost and duration of the exam - Availability - Contraindications with metal devices - Use of contrast - Limited accuracy on the LVOT pressure gradient - Low quality in arrhythmias of poor breath-holding ability	**Echocardiography:** - Initial screening - Risk stratification - Real-time and more accurate acquisition of the LVOT pressure gradient - Main imaging technique during follow-up	**Echocardiography:** - No LGE evaluation - Operator dependency
			**CT:** - Coronary visualization	**CT:** - Radiation exposure - Limited for LGE - Low quality in arrhythmias
			**Nuclear imaging:** - Evaluation of myocardial perfusion for microvascular ischemia	**Nuclear imaging:** - Radiation exposure - Limited for LGE
**Arrhythmogenic Cardiomyopathy**	- Evaluation of the right ventricle - Early recognition of fibro-adiposity - Differential diagnosis	- High cost and duration of the exam - Availability - Contraindications with metal devices - Use of contrast - Low quality in arrhythmias of poor breath-holding ability	**Echocardiography:** - Initial screening - Main imaging technique during follow-up	**Echocardiography:** - Limited for the right ventricle evaluation - Operator dependency
			**CT:** - Exclusion of coronary artery disease	**CT:** - Radiation exposure - No characterization of fibro-adiposity - Low quality in arrhythmias
**Restrictive** **Cardiomyopathy**	- Tissue differentiation (e.g., amyloidosis with characteristic LGE pattern) - Precise volumes measures - Differential diagnosis	- High cost and duration of the exam - Availability - Contraindications with metal devices - Use of contrast - Low quality in arrhythmias of poor breath-holding ability	**Echocardiography:** - Evaluation of diastolic function	**Echocardiography:** - Not specific for fibrosis - Operator dependency
			**Nuclear imaging:** - Useful in specific etiologies (PYP for amyloidosis) - Identification of inflammatory processes (e.g., sarcoidosis) - Therapeutic follow-up	**Nuclear imaging:** - Radiation exposure - Artifacts
**Left Ventricular Noncompaction**	- Best technique for defining non-compact trabeculature - Accurate volume measures - LGE for fibrosis - Differential diagnosis	- High cost and duration of the exam - Availability - Contraindications with metal devices - Use of contrast - Low quality in arrhythmias of poor breath-holding ability	**Echocardiography:** - Initial screening - Main imaging technique during follow-up	**Echocardiography:** - Limited ability to distinguish non-compact trabeculature from physiological hypertrophy
			**Cardiac CT:** - Good anatomical resolution to confirm trabeculature	**Cardiac CT:** - Does not evaluate functionality or tissue characterization - Low quality in arrhythmias
**Takotsubo Syndrome**	- Evaluation of edema - Accurate volumes measures	- High cost and duration of the exam - Availability - Contraindications with metal devices - Use of contrast - Low quality in arrhythmias of poor breath-holding ability	**Echocardiography:** - Accessibility - Useful for initial diagnosis - Main imaging technique during follow-up	**Echocardiography:** - Does not evaluate functionality or tissue characterization - Operator dependency
			**Nuclear imaging:** - Identification of transient ischemia	**Nuclear imaging:** - Does not evaluate functionality or tissue characterization

CT: computed tomography; CMR: cardiac magnetic resonance; LGE: late gadolinium enhancement; LVOT: left ventricular outflow tract obstruction; PYP: pyrophosphate.

## Abbreviations

The following abbreviations are used in this manuscript:

CMR	Cardiovascular magnetic resonance
CVD	Cardiovascular diseases
HF	Heart failure
EF	Ejection fraction
ECV	Extracellular volume
LGE	Late gadolinium enhancement
HFrEF	Heart failure with reduced EF
HFmrEF	Heart failure with mildly reduced EF
HFpEF	Heart failure with preserved EF
GLS	Global longitudinal strain
CRT	Cardiac resynchronization therapy
DCM	Dilated cardiomyopathy
SSFP	Steady-state free-precession
SCD	Sudden cardiac death
NT-proBNP	N-terminal pro B-type natriuretic peptide
HCM	Hypertrophic cardiomyopathy
ACM	Arrhythmogenic cardiomyopathy
ECG	Electrocardiography
RCM	Restrictive cardiomyopathy
CA	Cardiac amyloidosis
ATTR	Transthyretin amyloidosis
AL	Monoclonal immunoglobulin light-chain amyloidosis
LVNC	Left ventricular noncompaction
TS	Takotsubo syndrome
AFD	Anderson–Fabry disease
CT	Computed tomography
